# Effect of lateral hinge fractures for bone union and clinical outcomes following opening-wedge distal tibial tubercle osteotomy in comparison with opening-wedge high tibial osteotomy

**DOI:** 10.1186/s40634-023-00701-0

**Published:** 2023-12-14

**Authors:** Shuhei Otsuki, Kuniaki Ikeda, Takashi Ishitani, Yoshinori Okamoto, Hitoshi Wakama, Masashi Neo

**Affiliations:** Department of Orthopedic Surgery, Osaka Medical and Pharmaceutical University, 2-7 Daigakumachi Takatsuki, Osaka, 569-8686 Japan

**Keywords:** Open-wedge distal tibial tubercle osteotomy, Bone union, Hinge fracture, Clinical outcome

## Abstract

**Purpose:**

Although the effects of lateral hinge fractures (LHF) on bone union and clinical outcomes after opening-wedge high tibial osteotomy (OWHTO) have been established, the effects of LHF after opening-wedge distal tibial tubercle osteotomy (OWDTO) are unclear. We hypothesised that LHF after OWDTO would be associated with delayed bone union and result in poorer clinical outcomes than expected for LHF after OWHTO.

**Methods:**

This study enrolled 100 patients, with 50 OWDTO patients (18 men; mean age, 63.2 years) and 50 OWHTO patients compared based on the propensity score matched analysis. The effect of LHF on bone union was compared between the groups. Clinical outcomes were assessed using the Lysholm score and the Knee Injury and Osteoarthritis Outcome Score (KOOS) at the mean follow-up of 28 months.

**Results:**

There was no between-group difference in the incidence rate of LHF. However, the rate of bone union at the anterior flange in the presence of an LHF was significantly lower in the OWDTO (26%) than in the OWHTO (80%) 3 months postoperatively (*p* < 0.05), but no difference was observed 12 months postoperatively. The Lysholm score was significantly lower for patients with LHF following OWDTO than for OWDTO patients without LHF or OWHTO patients with/without LHF 3 and 12 months postoperatively (*p* < 0.001); Lysholm score and KOOS were not different at the final follow-up.

**Conclusions:**

LHF after OWDTO was associated with delayed bone union and poor clinical outcomes until 12 months. This information can guide decisions regarding the indications and the management of patients after OWDTO.

**Level of evidence:**

IV

## Introduction

Although favourable clinical outcomes have been reported with opening-wedge high tibial osteotomy (OWHTO) [1, 2], progression of patellofemoral osteoarthritis (OA) resulting from decreased patellar height following OWHTO remains an issue of concern [3–5], especially for older patients [6].

To minimize patellar height reduction, opening-wedge distal tibial tubercle osteotomy (OWDTO) was recommended so that OWHTO is performed below the tibial tuberosity; the descending incision begins distally in the frontal plane from the most anterior point of the transverse incision [3, 7, 8]. OWDTO is an alternative to OWHTO that provides similar favourable clinical outcomes while minimising unfavourable effects on the patellofemoral joint [3, 9, 10]. However, OWDTO is also associated with a risk of delayed union or non-union, just as lateral hinge fractures (LHF) are recognised as a risk factor in OWHTO [11–13].

LHF occurred in approximately 15–35% cases using X-rays and over 40% cases with CT scans of OWHTO [12, 14–16], and 27% of OWDTO [16], thus is not an uncommon complication for this procedure. LHF is a major cause of instability, leading to serious complications such as delayed bone union and correction loss [15] that might require additional surgery [17], and extended postoperative recovery, delayed return to daily activities, as well as a negative impact on patient-reported quality of life. However, the difference of bone union with LHF between OWHTO and OWDTO, and its effect on clinical outcomes remain unknown. Accordingly, this study aimed to evaluate the incidence of LHF and to compare clinical and radiographic outcomes between OWDTO and OWHTO using a propensity score-matched analysis. We hypothesised that LHF in OWDTO would be associated with delayed bone union and, thus, worse clinical outcomes compared to OWHTO.

## Materials and methods

### Statement of ethics

The methods of the study were approved by our institutional review board (No. 2022–023) and all patients provided written informed consent for the use of their data for research and publication.

### Study design

This was a retrospective comparative study between OWDTO and OWHTO for OA of the knee. From January 2017 to December 2020, a total of 209 knees (203 patients) were enrolled in this study.

Surgical indications for DTO and OWHTO were OA of the medial compartment of the knee, flexion contracture of < 10º, and deformity of the proximal tibia in patients with a body mass index (BMI) of less than 35 kg/m^2^, without diabetes mellitus or with well-controlled disease, and a non-smoker.

**Fig. 1:**
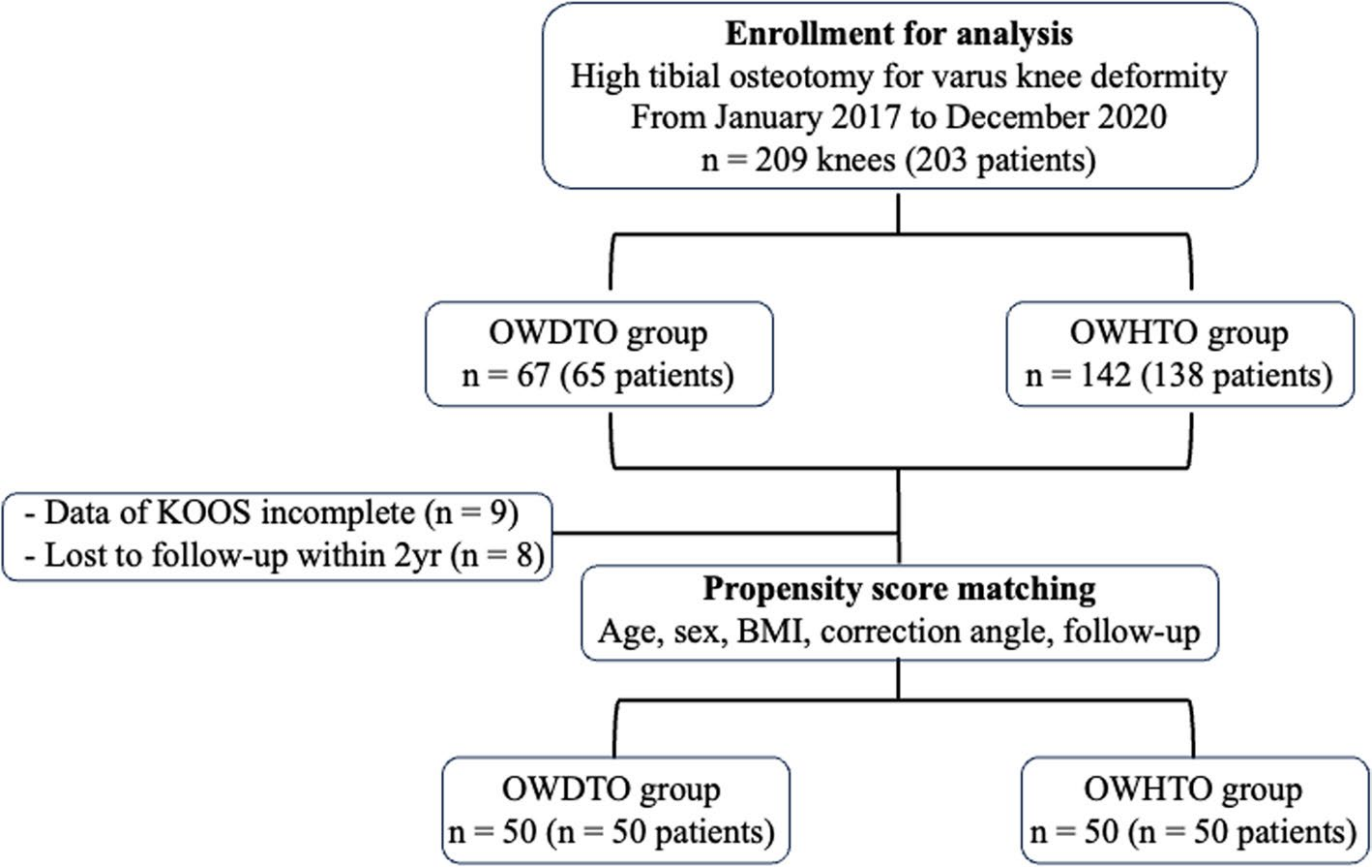
Patient selection flowchart. OWDTO, opening-wedge distal tubercle osteotomy; OWHTO, opening-wedge high tibial osteotomy. BMI, body mass index

OWDTO was performed on 67 knees of 30 men and 37 women, whose average age was 65.3 ± 8.4 years. For comparison, OWHTO was performed on 142 knees (54 men and 88 women) and the average age of the patients was 60.5 ± 9.4 years, which was significantly younger than that of OWDTO group (Table 1, *p* < 0.01). In the OWDTO group, 17 knees were excluded because 9 cases had incomplete data (KOOS) and 8 cases were lost to follow-up within 2 years; as a result, 50 knees were evaluated (Fig. 1). For the matching, the following clinically relevant potentially confounding covariates were chosen as previously proposed in the setting of alignment-correcting osteotomies [18]: age at surgery, sex, BMI, and postoperative correction angle. Fifty knees in the OWHTO group were enrolled via propensity score matching. The demographic data for both groups is presented in Table 1.

**Table 1:** Patient demographics

	OWDTO	OWHTO	*p* value
Total cases (*n* = 209)			
Age (years)	65.3 ± 8.4 (45–80)	60.5 ± 9.4 (40–75)	< 0.01
Knees (male / female patients)	67 (30/37)	142 (54/88)	0.35
BMI (kg/m^2^)	25.1 ± 3.9 (16.2–33.1)	24.8 ± 3.6 (16.5–31.1)	0.60
Correction angle (º)	8.9 ± 2.1 (6–13)	8.0 ± 2.7 (5–12)	0.26
Propensity score-matched cases (*n* = 100)			
Age (years)	63.2 (47–75)	63.3 (45–75)	0.55
Knees (male / female patients)	50 (18/32)	50 (18/32)	0.84
BMI (kg/m^2^)	25.1 ± 3.0 (17.8–31.5)	24.9 ± 2.8 (17.6–31.1)	0.63
Correction angle (º)	9.1 ± 1.5 (6–12)	8.9 ± 1.7 (7–12)	0.71

*BMI* body mass index, *OWDTO* opening-wedge distal tubercle osteotomy, *OWHTO* Opening-wedge high tibial osteotomy

### Surgical indication and procedure for OWDTO

Preoperative planning for OWHTO begins with the intended postoperative mechanical axis of the lower limb, which passes through the lateral tibial eminence as seen on the coronal view of whole-leg standing radiographs. For this study, the mechanical axis was analysed using digital planning software (TraumaCaD, BRAINLAB, Feldkirchen, Germany) with a picture archiving and communication system [5, 19]. Arthroscopy was performed routinely prior to surgery to evaluate the degree of cartilage degeneration and quantified using the International Cartilage Repair Society (ICRS) grading system [20]. The indication for OWDTO was an ICRS cartilage degeneration grade at the patellofemoral joint more than grade II; OWHTO was selected for ICRS grades 0 or I, with knee joint flexion contracture < 10°. Both HTOs were postoperative medial proximal tibial angle (MPTA) ≤ 95º.

**Fig. 2:**
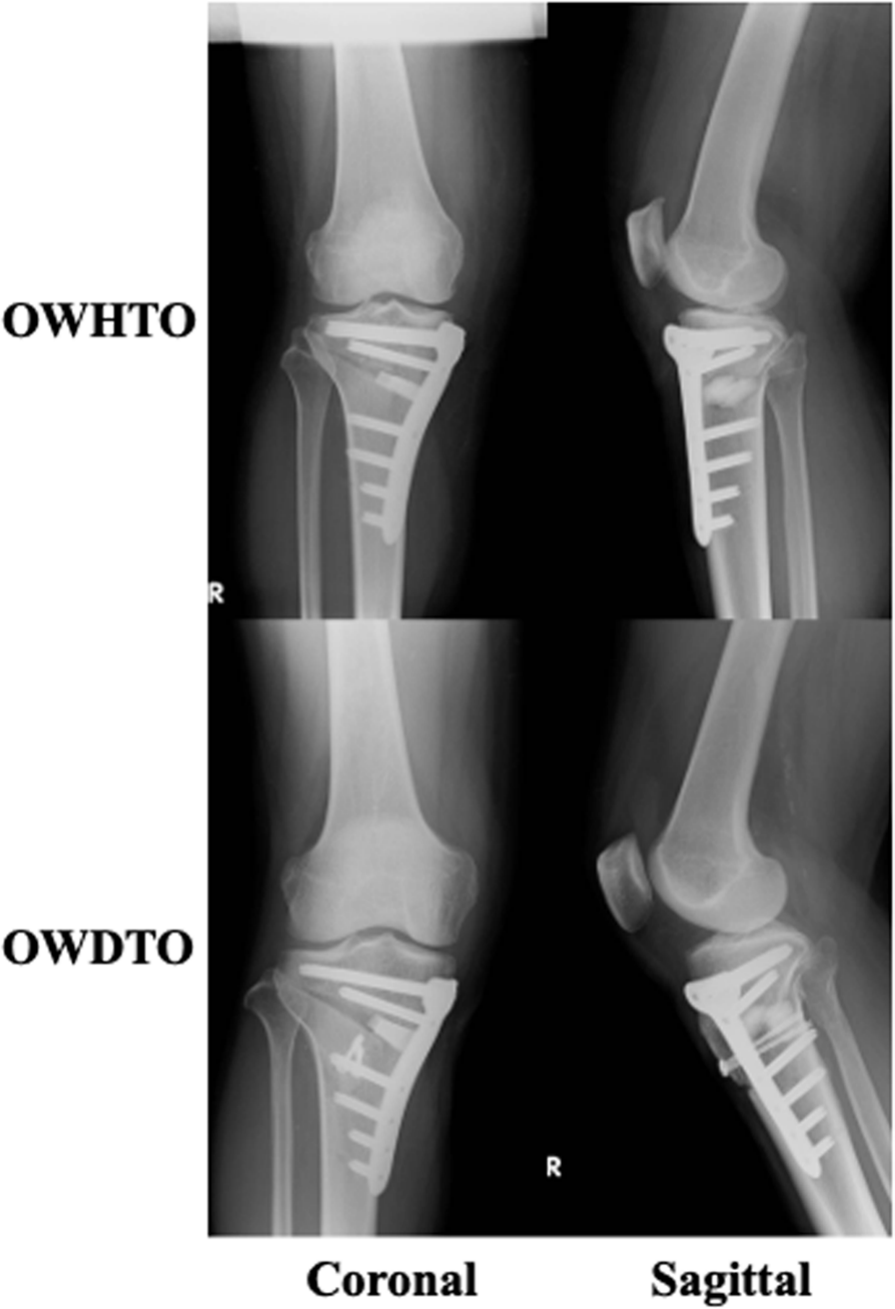
Representative X-ray of OWDTO and OWHTO

The surgical procedure for OWDTO has been described in detail previously [7] and is briefly summarised here. The descending osteotomy incision line was located 40 mm distal to the knee joint line, with two guidewires inserted in a parallel orientation and directed toward the tip of the head of the fibula. The hinge position was located 5 mm medial to the lateral cortical margin at the upper level of the proximal tibiofibular joint. The ascending osteotomy was then performed with a 15-mm thickness of the tibial tuberosity, oriented at 90º to the descending incision line. A short pin was placed at the hinge point, serving as a custom-designed compass for an arc osteotomy of the distal tibial tuberosity. The length of the arc osteotomy was measured from the hinge point to the end of the descending osteotomy, with the starting point set 20 mm distal to the descending incision line. Finally, the gap created during the descending osteotomy was filled using TriS plate and β-tricalcium phosphate wedge spacers (Olympus Termo Biomaterials, Tokyo, Japan), as previously reported [21]. The distal part of the tibial tuberosity was then fixed using one or two 4-mm cannulated screws (Asnis III; Stryker, MI, USA). Two screws were inserted medial distal and lateral proximal after arc osteotomy of the tibial tuberosity. Five patients (10%) were fixed using one screw because the size of the tibial tuberosity was small. Representative cases of X-rays of OWHTO and OWDTO are shown in Fig. 2. A single surgeon (S.O) performed the X-rays of all cases in this study.

Postoperatively, full weight-bearing was permitted on day 2 for both groups and range of motion exercises were started in the OWHTO group; however, OWDTO recipients were provided with a knee extension brace (NIPPON SIGMAX, Tokyo, Japan) for 2 weeks to prevent postoperative displacement at the tibial tuberosity. Muscle strengthening and passive range of motion exercises were initiated in both groups at week 2 postoperatively. All cases adopted this rehabilitation protocol in this study.

### Radiographic and clinical evaluation

**Fig. 3:**
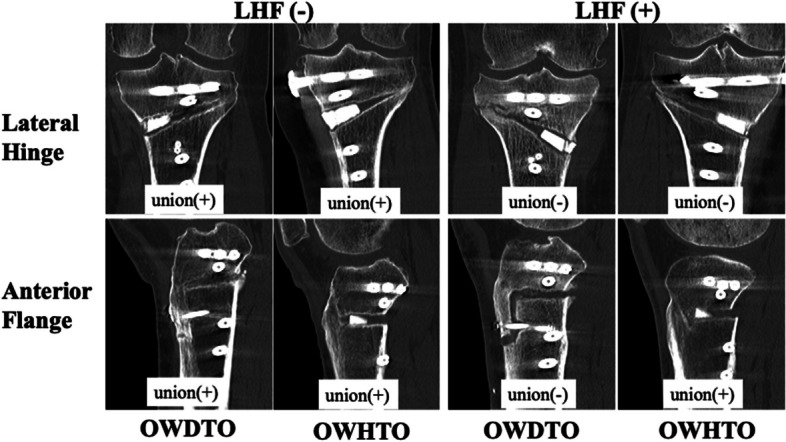
Coronal computed tomography images for the OWDTO and OWHTO patients. Coronal computed tomography images for the OWDTO and OWHTO groups, showing patients with and without a lateral hinge fracture. Bone union at the lateral hinge and anterior flange are shown for the OWDTO and OWHTO groups 3 months postoperatively. Bone union is observable in panels. OWDTO, opening-wedge distal tubercle osteotomy; OWHTO, opening-wedge high tibial osteotomy. *delayed bone union

Pre- and postoperative knee alignment were evaluated using the weight-bearing line ratio (WBLR), measured using whole-leg standing radiographs and by the femorotibial angle (FTA) and MPTA measured using coronal view radiographs. Posterior tibial slope (PTS) and patellar height were assessed using the Caton-Deschamps (CD) index on sagittal view radiographs with 30 degrees of knee flexion. The presence of LHF was evaluated using the Takeuchi classification on postoperative radiographs and computed tomography (CT) images at 3 months postoperatively [13]. Representative CT images of bone union 3 months postoperatively are shown in Fig. 3. Bone union was detected in both the OWDTO and OWHTO groups in the absence of LHF. However, delayed union was observed in the lateral hinge and anterior flange in the OWDTO with LHF, compared to the OWDTO group without LHF. Bone union was evaluated using the van Hemert classification at 12 months [22]. This classification was specific for the bone remodelling after high tibial osteotomy with bone substitute. All radiographic measurements were evaluated independently by two different co-authors, and interobserver and intraobserver reliability were over 0.90 in this study, as well as previously described [5]. Clinical outcomes were evaluated using the Lysholm score obtained preoperatively and at 3, 12 and 24 months postoperatively [23]. KOOS was compared preoperatively and at the final follow-up, which took place at an average of 28.8 (24–36) months.

### Statistical analysis

The primary outcome was the rate of bone union 12 months postoperatively, with the comparison between the OWDTO and OWHTO groups evaluated using the Wilcoxon test. Pre- to postoperative changes in lower limb alignment were compared between the two groups using the Wilcoxon test. The incidence rate of LHF and the association between LHF and delayed union were compared between the two groups using the chi-squared test. The association between LHF and the Lysholm score was compared between groups using the chi-squared test. Analyses were performed using JMP Pro (version 15.1.0; SAS Institute), with significance set at a *p*-value of < 0.05. A power analysis was performed using bone union as the primary outcome (G*power, version 3.1.9.2, Dusseldorf, Germany). For an α-value of 0.05 and power of 0.8, a total of at least 52 knees was required for evaluation of between-group differences.

## Results

**Fig. 4:**
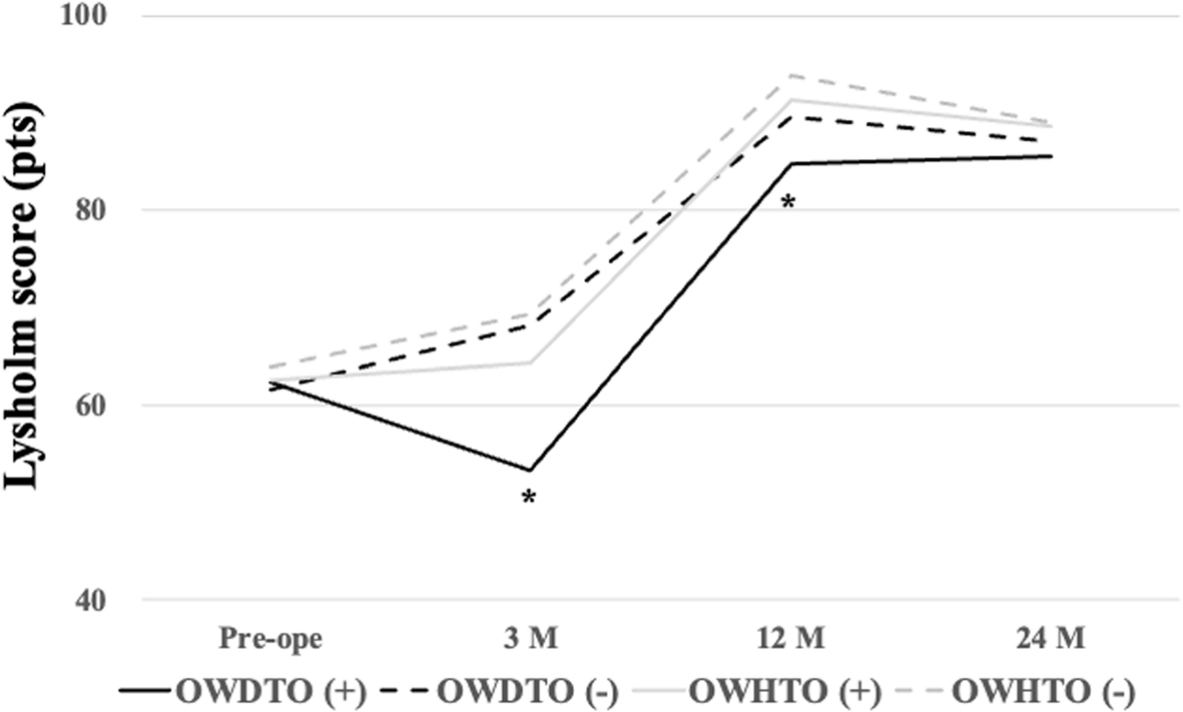
Comparison of clinical outcomes, measured using the Lysholm score, between the OWDTO and OWHTO groups. The score was lower for patients with a lateral hinge fracture (LHF) in the OWDTO compared to all other groups (OWDTO without LHF, OWHTO with and without an LHF) 3 and 12 months postoperatively (*, *p* < 0.01). OWDTO, opening-wedge distal tubercle osteotomy; OWHTO, opening-wedge high tibial osteotomy; OWHTO (-), OWHTO without LHF; OWHTO ( +), OWHTO with LHF

There were no significant differences between the groups in terms of lower limb alignment indices (WBLR, FTA, MPTA, and PTS). Regarding within-group differences, there was a significant pre- to postoperative decrease in the CD index for the OWHTO group (Table 2, *p < 0.001), and lower in comparison to OWDTO (Table 2, *p* < 0.01). LHF occurred in 47 patients and all LHF were Takeuchi classification type 1 and did not delay the rehabilitation protocol. LHF occurred in 27 patients (54%) with OWDTO procedures, which was not significantly different from its occurrence in 20 patients (40%) in OWHTO procedures and the ratio of sex in LHF was not significantly different (Table 3). Age, BMI and correction angle were not significantly different in both groups. However, bone union at the anterior flange was significantly delayed in the OWDTO group with LHF (26%) compared to the OWDTO group without LHF (78%), and the OWHTO groups with LHF (80%) and without LHF (87%) at postoperative 3 months (Table 4; *p < 0.001). Regarding complications, there were two fractures and one screw-loosening at the tibial tuberosity in the OWDTO group with LHF and one fracture in the OWDTO group requiring additional surgery for fixation at the tibial tuberosity at 2 weeks postoperatively. An additional two cases were delayed in starting the rehabilitation program, especially the range of motion (ROM) exercises for 1 month. However, loss of correction angle was not detected in all cases, and bone union 12 months postoperatively was not significantly different between the OWDTO and OWHTO groups with and without LHF (Table 5). The postoperative Lysholm score was significantly lower for the OWDTO group with LHF compared to the OWDTO group without LHF and the OWHTO groups with and without LHF at 3 and 12 months postoperatively (Fig. 4; *p < 0.001), whereas it was not significantly different at postoperative 24 months and for the KOOS at the final follow-up (Fig. 4, Table 6).

**Table 2:** Change in knee alignment measures from baseline to post-OWHTO and OWDTO

	OWDTO (*n* = 50)	OWHTO (*n* = 50)	*p* value
WBLR (%)			
pre	19.8 ± 10.9	20.4 ± 10.2	0.80
post	59.2 ± 8.1	58.6 ± 2.9	0.79
FTA (°)			
pre	181.4 ± 2.4	182.0 ± 2.9	0.26
post	171.1 ± 2.6	171.6 ± 2.3	0.58
MPTA (°)			
pre	83.4 ± 2.1	84.0 ± 2.9	0.22
post	91.6 ± 2.0	91.7 ± 2.2	0.51
PTS (°)			
pre	6.9 ± 2.3	7.6 ± 2.8	0.22
post	8.3 ± 3.0	9.0 ± 2.9	0.21
CD index			
pre	0.95 ± 0.17	0.99 ± 0.08	0.26
post	0.98 ± 0.11	0.88 ± 0.10^*^	< 0.01

*WBLR* weight-bearing line ratio, *FTA* femorotibial angle, *MPTA* medial proximal tibial angle, *PTS* posterior tibial slope, *CD index* Caton-Deschamps index

^*^p < 0.001 within-group change

**Table 3:** Incidence of lateral hinge fracture with OWDTO and OWHTO

	OWDTO (*n* = 50)	OWHTO (*n* = 50)	*p* value
LHF ( +)	27 (13/14)	20 (10/10)	0.90
LHF (-)	23 (5/18)	30 (8/22)	0.68

Number of knees and sex of the patients (male/female) are shown. Between-group *p* value evaluated using the chi-squared test (χ2), *p* = 0.16

*LHF* lateral hinge fracture, *OWDTO* opening-wedge distal tubercle osteotomy, *OWHTO* opening-wedge high tibial osteotomy

**Table 4:** Incidence rate of bone union with and without presence of a lateral hinge fracture 3 months postoperatively

Bone union	LHF (-)		LHF ( +)	
	OWDTO (*n* = 23)	OWHTO (*n* = 30)	OWDTO (*n* = 27)	OWHTO (*n* = 20)
Anterior	18 (78%)	26 (87%)	7 (26%)*	16(80%)
Lateral	23 (100%)	30 (100%)	11 (41%)	9 (45%)

Between-group *p*-value evaluated using the chi-squared test (χ2); LHF (-), absence of a lateral hinge fracture; LHF ( +), presence of a lateral hinge fracture

*OWDTO* opening-wedge distal tibial tubercle osteotomy, *OWHTO* opening-wedge high tibial osteotomy

^*^p < 0.05 versus an OWDTO in the corresponding group

**Table 5:** Bone union achieved at postoperative 12 months, according to the van Hemert classification phase (0–5)

van Hemert Phase	2	3	4	5	*p* value
LFH (-)					
OWDTO (*n* = 23)	1	2	5	15	
OWHTO (*n* = 30)	0	5	11	14	0.29
LFH ( +)					
OWDTO (*n* = 27)	6	4	10	7	
OWHTO (*n* = 20)	1	9	6	4	0.09

Between-group *p* value evaluated using the chi-squared test (χ2); LHF (-), absence of a lateral hinge fracture; LHF ( +), presence of a lateral hinge fracture

*OWDTO* opening-wedge distal tibial tubercle osteotomy, *OWHTO* opening-wedge high tibial osteotomy

**Table 6:** Comparison of Knee Injury and Osteoarthritis Outcome Score between OWDTO and OWHTO associated with lateral hinge fracture

	OWDTO			OWHTO		
	LFH ( +)	LFH (-)	*P* value	LFH ( +)	LFH (-)	*P* value
Preoperative						
Symptom	62.8 ± 19.5	69.6 ± 18.6	0.43	69.0 ± 16.8	66.3 ± 14.3	0.57
Pain	56.1 ± 17.6	50.6 ± 18.5	0.28	67.7 ± 11.7	62.3 ± 12.5	0.16
ADL	71.5 ± 15.2	75.7 ± 14.8	0.43	77.5 ± 10.6	71.8 ± 14.2	0.15
Sports	36.1 ± 21.7	41.9 ± 30.2	0.60	35.5 ± 14.4	36.8 ± 21.1	0.82
QoL	35.6 ± 22.3	38.3 ± 24.7	0.94	32.2 ± 15.9	33.8 ± 19.3	0.78
Final Follow-up						
Symptom	75.4 ± 16.1	79.2 ± 14.0	0.24	75.6 ± 15.0	83.0 ± 13.3	0.09
Pain	75.9 ± 14.7	78.3 ± 17.2	0.32	76.2 ± 16.1	82.3 ± 11.1	0.14
ADL	82.7 ± 11.8	85.2 ± 11.3	0.31	83.6 ± 9.6	88.4 ± 7.3	0.07
Sports	50.0 ± 26.5	59.1 ± 25.6	0.25	57.9 ± 23.4	67.6 ± 22.0	0.16
QoL	56.5 ± 20.5	62.1 ± 22.5	0.16	56.8 ± 21.3	68.0 ± 19.5	0.08

*p* value using the Wilcoxon test. OWDTO; open wedge distal tubercle osteotomy, OWHTO; open wedge high tibial osteotomy; LHF ( +), presence of a lateral hinge fracture; LHF (-), absence of a lateral hinge fracture

*ADL* activities of daily living, *QoL* quality of life

## Discussion

The most important findings of this study were that LHF occurred after OWDTO as well as OWHTO; however, LHF after OWDTO was detected by delayed bone union especially at the anterior flange, with poorer clinical outcomes.

**Fig. 5:**
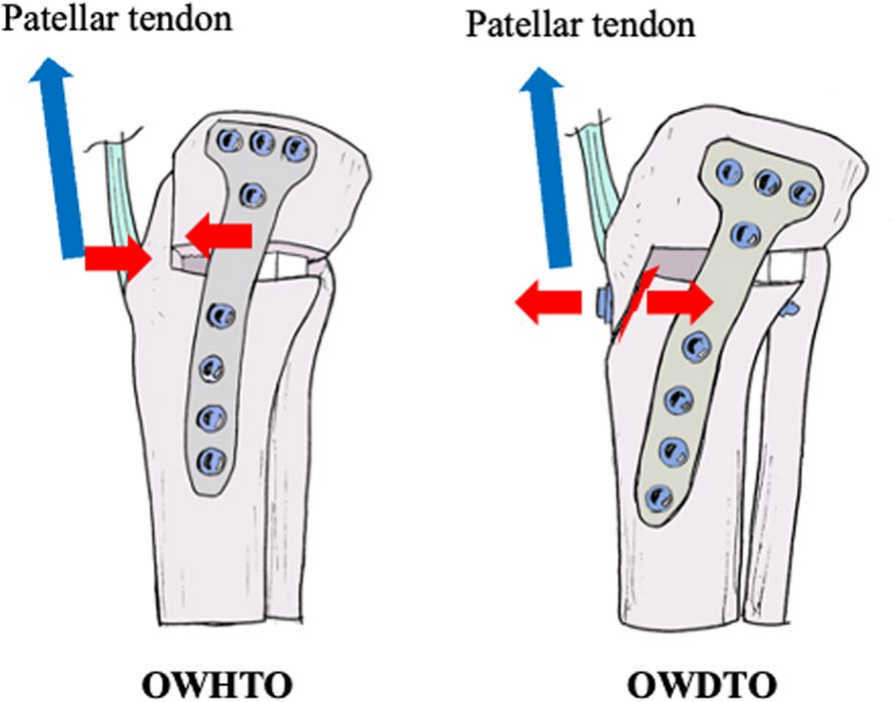
Mechanism leading to a delayed union at the anterior flange. The anterior flange is compressed by the patellar tendon during knee flexion following OWHTO. By comparison, the anterior flange is pulled apart by the patellar tendon during knee motion after OWDTO. A lateral hinge fracture (LHF) causes greater instability, especially with OWDTO. OWDTO, opening-wedge distal tubercle osteotomy; OWHTO, opening-wedge high tibial osteotomy

LHF frequently occur in the area surrounding knee osteotomy sites. These hinge fractures have been classified by Takeuchi et al. for OWHTOs [13]. Complications of LHF, including delayed bone union, loss of alignment correction, and sustained lateral pain, have previously been reported [13, 24–26]. In the current study, delayed union of the anterior flange was associated with LHF after OWDTO but not after OWHTO, reflecting differences in the biomechanical effects of OWDTO compared to those of OWHTO (Fig. 5). Specifically, OWDTO produces less compression across the osteotomy site compared to OWHTO, with the patellar tendon producing traction on the anterior flange, leading to lower overall mechanical stability [4]. Although a stable fixation is reached, the postoperative rehabilitation protocol for the OWDTO needs no adjustments due to the new technique [3], the occurrence of LHF following OWDTO results in a high magnitude of stress on the plate and screw fixation, as no bony or tendon connection remains at the osteotomy site. Furthermore, knee joint ROM exercises induce additional traction forces at the tibial tuberosity via the patellar tendon. Accordingly, care should be taken in determination of when to initiate ROM exercises post-OWDTO owing to the risk of tibial tuberosity fracture and screw loosening, especially in patients with high BMI or osteoporosis.

Previous studies have reported similar clinical outcomes after OWHTO with and without LHF [13, 27, 28]. Our study, comparing outcomes between patients with and without LHF after OWHTO and OWDTO, identified lower Lysholm scores, as the measure of clinical outcomes, until 12 months postoperatively among patients who developed LHF after OWDTO. This measure resulted in a longer time taken for returning to activities of daily living and work. Moreover, one patient in this group required additional surgery for treatment of unstable tibial tuberosities.

Additional surgical techniques might be required to prevent the LHF [29], for example, protective K-wire has been shown to significantly decrease the risk of hinge fractures [30, 31]. To improve clinical outcomes of LHF after OWDTO, previous studies have suggested the use of gap fillers created from autologous iliac crest; allogenic bone grafts; or bone graft substitutes such as β-TCP, hydroxyapatite and, autologous osteophytes [32, 33]. Supplementing stability of the osteotomy site with percutaneous lag screws may also improve stability in cases of LHF after OWDTO [34]. The limitations of our study need to be acknowledged. Foremost is its retrospective design, and propensity score-matched analysis was used to account for the differences in the patients’ background characteristics between the OWDTO and OWHTO groups. Second, the surgical indications and postoperative rehabilitation program for OWDTO and OWHTO differed, particularly in terms of patellofemoral cartilage degeneration, which was more severe in the OWDTO group than in the OWHTO group, and thus may have influenced the process of postoperative bone union and clinical outcomes including the Lysholm score and KOOS.

## Conclusion

LHF following OWDTO increased the risk of delayed bone union, especially at the anterior flange, and poor clinical outcomes were observed until 12 months postoperatively compared to OWHTO. Rehabilitation programs should be judiciously prescribed when LHF occurs following OWDTO.

## Abbreviations

BMI: Body mass index
CD index: Caton-Deschamps index
CT: Computed tomography
FTA: Femorotibial angle
ICRS: International Cartilage Repair Society
KOOS: Knee Injury and Osteoarthritis Outcome Score
LHF: Lateral hinge fractures
MPTA: Medial proximal tibial angle
OA: Osteoarthritis
OWDTO: Opening-wedge distal tibial tubercle osteotomy
OWHTO: Opening-wedge high tibial osteotomy
PTS: Posterior tibial slope
ROM: Range of motion
WBLR: Weight-bearing line ratio
